# Revealing the Flavor and Metabolite Differences of Chinese Sweet Rice Wine Fermented with Diverse Rice Varieties Using GC-IMS and UPLC-MS/MS

**DOI:** 10.3390/foods15122137

**Published:** 2026-06-13

**Authors:** Qi Zheng, Wenhui Tian, Ling Yue, Qiulian Kong, Haihong Wang, Zhijun Chen, Yi Zhang, Chunfang Wang, Songheng Wu, Weiqiang Yan, Shujun Wu

**Affiliations:** 1 Crop Breeding & Cultivation Research Institute, Shanghai Academy of Agricultural Sciences, 1000 Jinqi Road, Fengxian District, Shanghai 201403, China; zhengqi@saas.sh.cn (Q.Z.);; 2 Shanghai Shuneng Irradiation Technology Co., Ltd., Shanghai Academy of Agricultural Sciences, 255 Maoyuan Road, Fengxian District, Shanghai 201403, China

**Keywords:** Chinese sweet rice wine, metabolic pathway, metabolites, volatile compounds, sensory evaluation

## Abstract

Japonica rice offers high cost-effectiveness and yield, with the potential to replace glutinous rice in Chinese sweet rice wine (CSRW) brewing. It can be classified into aromatic and non-aromatic types, but whether different varieties cause flavor and metabolite differences in CSRW remains unclear. In this study, glutinous rice (GR), three aromatic japonica varieties (CS-217, HXR-450, SXJ-1018), and two non-aromatic varieties (TA-1, HR-1212) were used as raw materials. The qualities of different CSRWs were evaluated through physicochemical indices, sensory evaluation, phenolic and flavonoid contents, antioxidant capacities, HS-GC-IMS, and UPLC-MS/MS. The results showed that CS-217 displayed the highest total acid content, along with excellent overall sensory evaluation, total phenolic content, and antioxidant capacity. A total of 28 VOCs were identified by HS-GC-IMS, among which 13 compounds with VIP ≥ 1, including butyl isobutyrate, butyl acetate, and ethyl pentanoate, were identified as key flavor discriminant factors. Additionally, 2501 non-volatile metabolites were identified, and five key metabolic pathways were revealed. These pathways synergistically regulate CSRW flavor and nutritional quality. Different japonica rice varieties exhibited respective advantages in CSRW quality indicators, providing a basis for the diversification of raw materials in CSRW production.

## 1. Introduction

Chinese sweet rice wine (CSRW) is a traditional Chinese alcoholic beverage that has been an essential part of the cultural and spiritual life of the Chinese people for thousands of years [1]. Together with beer and wine, it is recognized as one of the three most ancient alcoholic beverages worldwide. It is favored by consumers for its unique flavor, mild taste, delicate aroma, rich nutritional composition, and low alcohol content [2]. At present, the production of CSRW has gradually transitioned from traditional household workshops to industrial and standardized manufacturing. The distinctive characteristics of this low-alcohol beverage arise from the interplay of enzymatic activities and microbial metabolism during production, a process that not only enhances the sensory quality of CSRW but also improves its nutritional value. Studies have shown that CSRW is rich in oligosaccharides, peptides, amino acids, vitamins, and minerals, and exhibits multiple health benefits, including antioxidant, hypoglycemic, hypolipidemic, and antihypertensive effects, as well as immune enhancement and improvement of intestinal health [2,3,4].

In CSRW brewing, glutinous rice (*Oryza sativa* var. glutinosa) is the most widely used raw material due to its easy gelatinization and high amylopectin content, which contribute to achieving the desired sweetness after fermentation [5]. With the continuous development of the brewing industry and the trend toward a younger consumer market, the CSRW market has shown rapid growth. However, as consumers’ pursuit of health, nutrition, and diverse tastes is increasing, CSRW has transitioned from a traditional food product into an emerging consumer category, giving rise to new rice wine products such as buckwheat rice wine [6], brown glutinous rice wine [7], and red rice wine [8]. Japonica rice (*Oryza sativa* var. japonica), characterized by lower viscosity and water absorption, exhibits an amylose structure similar to that of glutinous rice, while offering greater cost-effectiveness and higher yield [9]. Similar fermented rice beverages, such as Japanese sake and Korean Makgeolli, utilize japonica rice in their production [10]. Nevertheless, the flavor and taste of CSRW brewed from different raw materials may differ significantly; therefore, identifying their flavor compounds and metabolites is crucial for optimizing the fermentation process and selecting appropriate raw materials for rice wine production.

The essence of sweet rice wine brewing lies in the complex processes of microbial growth, proliferation, and metabolism. During these processes, microorganisms utilize grains to produce ethanol and various flavor compounds [11]. To ensure product quality, modern CSRW production mainly employs pure cultures of *Rhizopus arrhizus* as the fermentation starter. Given that the microbial strain is relatively fixed, the differences in raw materials become a crucial factor shaping the flavor characteristics and quality of CSRW. The influence of raw materials on wine flavor is primarily manifested in two aspects. First, the characteristic flavor compounds of raw materials are directly leached or enzymatically released into the wine during fermentation, forming “raw material aroma”. Second, differences in the nutritional composition of raw materials can indirectly affect the growth and metabolic activities of microorganisms, thereby altering the final flavor of the wine [12]. Furthermore, proteins in raw materials are hydrolyzed by enzymes during fermentation to generate peptides and free amino acids, which not only serve as nutrients in rice wine but also act as precursors for the Maillard reaction and Strecker degradation, directly participating in the formation of aroma compounds [13,14]. Compared with glutinous rice, japonica rice generally has a higher protein content and can be used to produce rice wine with higher amino acid levels [15].

Based on aroma, rice can be classified into two categories: aromatic and non-aromatic, and their sensory characteristics are primarily determined by volatile compounds [16]. Aromatic rice is renowned for its appealing fragrance and has gained increasing popularity in rice-consuming countries, accounting for approximately 15–18% of the global rice trade [17]. To date, more than 250 volatile compounds have been identified in rice [18]. Notably, some rice-derived volatiles, such as hexanal, octanal, nonanal, ethyl acetate, 1-hexanol, ethyl butanoate, and ethyl 3-methylbutanoate, are also key aroma compounds in CSRW [6,9,10]. Whether these aroma compounds in aromatic rice can influence the flavor and quality of CSRW has not yet been reported.

Therefore, this study aimed to reveal the compositional and flavor differences in CSRW brewed from glutinous rice, aromatic japonica rice, and non-aromatic japonica rice. By determining the physicochemical indices and antioxidant capacities of CSRW produced from different raw materials, and employing headspace gas chromatography-ion mobility spectrometry (HS-GC-IMS) and ultra-performance liquid chromatography-tandem mass spectrometry (UPLC-MS/MS) techniques combined with multivariate statistical analysis, the volatile organic compounds (VOCs) and non-volatile metabolites in different CSRW samples were identified, and the key metabolites responsible for the differences were clarified. The findings of this study provide critical reference data and technical support for the development of CSRW products.

## 2. Materials and Methods

### 2.1. Materials

Glutinous rice (GR, protein 6.6%, amylose 1.6%, total starch 80.3%) was procured from a local supermarket in Shanghai, China. Three types of aromatic japonica rice, including Chongshang217 (CS-217, protein 9.3%, amylose 9.0%, total starch 73.3%), Huxiangruan450 (HXR-450, protein 7.6%, amylose 9.1%, total starch 71.8%), and Songxiangjing1018 (SXJ-1018, protein 7.6%, amylose 9.8%, total starch 74.5%), as well as two types of non-aromatic japonica rice, specifically Taian No.1 (TA-1, protein 6.9%, amylose 8.8%, total starch 73.7%) and Huruan1212 (HR-1212, protein 7.1%, amylose 8.3%, total starch 72.1%), were obtained from Crop Breeding & Cultivation Research Institute, Shanghai Academy of Agricultural Sciences, Shanghai, China. All japonica rice was harvested in 2024. For each rice variety, three independent bags were collected from separate plots within the same growing base. The commercially available Jiuqu, specifically produced for CSRW fermentation, was purchased from Angel Yeast Company in Yichang, China.

### 2.2. CSRW Fermentation

All raw materials, including rice varieties and jiuqu, were obtained from the same supplier to ensure the uniformity across the batches and consistency of CSRW production. The proportion of brewing raw materials remained consistent across all treatments, ensuring that differences in physicochemical properties, volatile organic compounds, and metabolites could be primarily attributed to the variation in rice varieties. The brewing process was performed in a temperature-controlled environment to ensure experimental reproducibility. The brewing procedure of CSRW was carried out according to the method described by Xiong et al. [6], with minor modifications. Briefly, 100 g of rice was soaked in distilled water at room temperature for 12 h. After draining, the rice was steamed in a steamer for 30 min until fully cooked and well loosened. The steamed rice was then spread evenly on a pre-cleaned, disinfected, and aseptic platform and naturally cooled to approximately 30 °C. Subsequently, a commercial Jiuqu specifically produced for CSRW fermentation (0.4% of rice weight) and 75 mL of sterile water were added and thoroughly mixed with the cooled rice. The mixture was transferred into a pre-sterilized round glass jar (500 mL) and flattened, followed by manually punching a hole with a diameter of about 2 cm in the center. The jar was then sealed with its original lid and placed in an incubator for fermentation. Fermentation was conducted at a constant temperature of 30 °C for 72 h. After fermentation, the CSRW sample was filtered and pasteurized at 75 °C for 20 min to obtain the final product. Three replicates were set up for CSRW brewing using each rice variety.

### 2.3. Physicochemical Analyses

The contents of total sugar and total acid were determined according to the previous studies [19,20]. The total sugar was determined by a Fehling reagent method. Briefly, the sample was subjected to acid hydrolysis to convert polysaccharides and disaccharides into reducing sugars. After cooling and neutralization, the solution was diluted to a constant volume and tested according to the Fehling reagent method. The total acid was measured using the titratable acidity method. The volume of NaOH solution (0.1 mol/L) required for titration was recorded. The pH was measured using a pH meter (Shanghai Rex Instrument Co., Ltd., Shanghai, China). The alcohol content was checked using an alcohol meter (Anton Paar GmbH, Graz, Austria).

### 2.4. Sensory Evaluation

The sensory evaluation panel consisted of two professional huangjiu tasters and eight trained evaluators. All members (aged 21–60) were in good health with no sensory impairments. The tasters did not know the treatment of the samples beforehand. A total of 50 mL sample was placed in a clean tasting glass, with approximately 5 mL per panelist. The relevant characteristics of the rice wine were perceived through appearance, aroma, and taste, followed by scoring the overall harmony based on these characteristics. The sensory attributes evaluated included: appearance (hue consistent with raw materials), aroma (distinctive clean fragrance characteristic of rice wine), taste (balanced sweetness and acidity with pleasant palatability), and overall harmony (balance of appearance, aroma, and taste, persistence, and aftertaste). The intensity was scored on a scale of 1 (worst) to 10 (best). The evaluation employed a weighted scoring system based on percentage allocation: appearance (10%), aroma (20%), taste (30%), and overall harmony (40%). The total score was calculated on a 10-point scale, where each attribute’s contribution was determined by its designated weight [6].

### 2.5. Detection of Total Phenols and Flavonoid Contents

The total phenolic content was determined using the Folin–Ciocalteu method according to a previous study, with some modifications [21]. Briefly, 100 μL of CSRW sample was mixed with 2.5 mL of 10% Folin–Ciocalteu reagent and allowed to react for 3 min. Subsequently, 2 mL of 7.5% sodium carbonate (Na_2_CO_3_) solution was added to the mixture. After incubation at 25 °C for 2 h in the dark, the absorbance was measured at 760 nm. Gallic acid was used as the standard for calibration curve preparation.

The total flavonoid content in CSRW samples was determined using the aluminum nitrate colorimetric method [6]. Specifically, 3 mL of 70% ethanol solution, 1 mL of sample, and 0.5 mL of 5% sodium nitrite solution were mixed, followed by a 6 min incubation. Then, 0.4 mL of 10% aluminum nitrate solution was added, and the mixture was allowed to stand for another 6 min. Finally, 0.8 mL of 4% sodium hydroxide solution was added, and the volume was adjusted to 10 mL with distilled water. After thorough mixing and 15 min of incubation, the absorbance was measured at 510 nm. Rutin was used as the standard to establish the calibration curve for quantifying total flavonoid content.

### 2.6. Determination of Antioxidant Activity

The antioxidant activity was determined by Ferric ion reducing antioxidant power (FRAP), ABTS free radical scavenging rate, and DPPH free radical scavenging rate using commercial assay kits (Fujian Herui Biotechnologies Co., Ltd., Fuzhou, China) in accordance with the manufacturer’s instructions.

### 2.7. HS-GC-IMS Analysis

The VOCs of the CSRW sample were analyzed by HS–GC-IMS according to Zheng et al. with minor modifications [22]. In brief, 2.0 mL of the sample was added to a 20 mL headspace bottle, then incubated at 60 °C and 500 rpm for 15 min. Subsequently, 0.5 mL of the headspace gas was injected into the heated needle port at 85 °C. The separation was conducted on an MXT-WAX capillary column (30 m × 0.53 mm × 1 μm, G.A.S., Dortmund, Germany) at 60 °C. Nitrogen gas (99.99% purity) was used as the carrier gas, with the flow rate programmed as follows: 2 mL/min for 0–2 min, 2 mL/min to 15 mL/min for 2–10 min, 15 mL/min to 100 mL/min for 10–20 min, 100 mL/min to 150 mL/min for 20–30 min. The resulting ions were driven to the drift tube (9.8 cm in length), which was operated at a constant temperature of 45 °C and a voltage of 5 kV, with a drift gas flow rate of 150 mL/min. The total analysis time was 30–60 min. The retention index (RI) of each volatile compound was calculated using n-ketone C4–C9 (Sigma-Aldrich Trading Co., Ltd., Shanghai, China) as a reference. Characterization was performed by comparison with the retention index (RI) and drift time (DT) of the standards in the GC-IMS database. All analyses were repeated three times. Due to the absence of external calibration or an internal standard, the signal intensities were used for the relative quantification of VOCs and for the calculation of relative odor activity values (ROAV) [22,23].

### 2.8. UPLC-MS/MS Analysis

The analysis was performed using an ACQUITY UPLC I-Class plus system (Waters, Milford, MA, USA) coupled with a Q-Exactive mass spectrometer (Thermo Fisher Scientific, Waltham, MA, USA) equipped with a heated electrospray ionization (H-ESI) source. Metabolic profiling was conducted in both electrospray ionization (ESI) positive and negative ion modes. Chromatographic separation was achieved on an ACQUITY UPLC HSS T3 column (100 mm × 2.1 mm, 1.8 μm). Mobile phase A consisted of water containing 0.1% formic acid, and mobile phase B was acetonitrile. The gradient elution program was as follows: 0–2 min, 5% B; 2–4 min, 5–30% B; 4–8 min, 30–50% B; 8–10 min, 50–80% B; 10–14 min, 80–100% B; 14–15 min, 100% B; 15–15.1 min, 100–5% B; 15.1–16 min, 5% B. The injection volume, column temperature, and flow rate were set at 3 μL, 45 °C, and 0.35 mL/min, respectively. The mass spectrometer was operated with spray voltages of 3800 V in positive ion mode and −3200 V in negative ion mode. Spectra were acquired over a scan range of 70–1050 *m*/*z*, with a resolution of 70,000 for full MS scans and 17,500 for high-energy collisional dissociation (HCD) MS/MS scans. Collision energies were set at 10, 20, and 40 electron volts (eV). A pooled quality control (QC) sample was prepared by mixing aliquots of all experimental samples. The QC sample was used to equilibrate the LC-MS system prior to sample analysis and to monitor system stability throughout the analytical sequence.

The raw data obtained were imported into XCMS software (v4.5.1) for baseline filtering, peak detection, integration, and retention time alignment. Metabolite identification was performed based on multiple dimensions, including retention time (RT), exact mass, MS/MS fragmentation patterns, and isotopic distribution, using public databases such as the Human Metabolome Database (HMDB), Lipidmaps (v2.3), METLIN, and the self-built database LuMet-Animal 3.0. No authentic standards were used. A scoring system was used, with a maximum total score of 80 points allocated as follows: 20 points for accurate precursor ion mass matching, 20 points for MS/MS fragmentation pattern matching, 20 points for isotopic distribution matching, and 20 points for RT matching. Identifications scoring below 36 were removed. Based on RT deviation (±0.3 min) and fragmentation score (out of 80), remaining identifications were classified into four levels: Level 1 (RT deviation ≤ ±0.3 min and fragmentation score ≥ 45); Level 2 (RT deviation ≤ ±0.3 min and fragmentation score < 45); Level 3 (fragmentation score ≥ 45); Level 4 (fragmentation score < 45). Only Level 1 and 2 metabolites were kept. Additionally, total peak area normalization was applied to the data to ensure comparability across samples and metabolites.

### 2.9. Statistical Analysis

Statistical analyses and bar chart plotting were performed using GraphPad Prism 8 (GraphPad Software, CA, USA). One-way analysis of variance (ANOVA) followed by Tukey’s HSD was used to determine significant differences among groups (*p* < 0.05). All experimental data were expressed as mean ± standard deviation. HS-GC-IMS data were visualized and analyzed using Laboratory Analytical Viewer (LAV, G.A.S., Dortmund, Germany). Principal component analysis (PCA) was performed using an online tool (https://cloud.oebiotech.com/), and partial least squares discriminant analysis (PLS-DA) was conducted using Simca 14.1 (Umetrics, Umeå, Sweden). For LC-MS/MS data, PCA, volcano plot, Kyoto Encyclopedia of Genes and Genomes (KEGG) pathway enrichment analysis, and Metabolomics Pathway Analysis (MetPA) were performed using an online tool (https://cloud.oebiotech.com/).

## 3. Results and Discussion

### 3.1. Physicochemical Characteristics and Sensory Analysis

**Figure 1:**
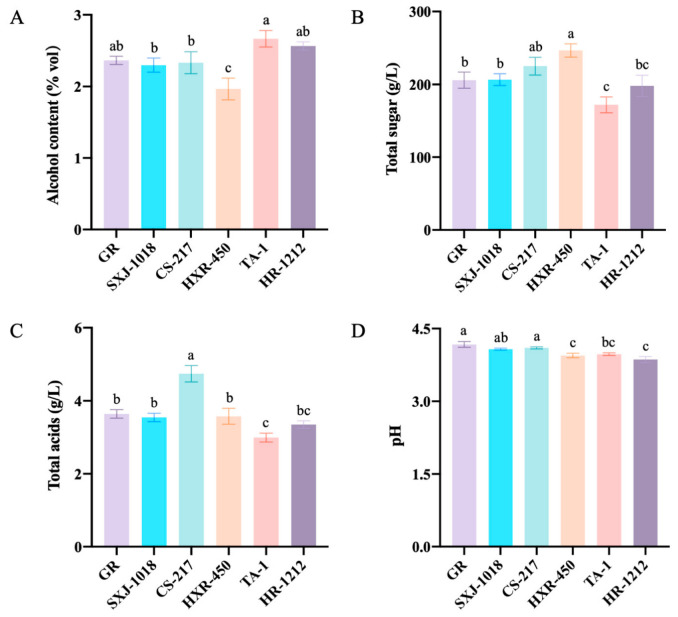
Alcohol content (**A**), total sugars (**B**), total acids (**C**), and pH (**D**) in different CSRW. Error bars represent the standard deviation of the mean scores. Different letters mean significant differences between groups (*p*-value < 0.05), data are presented as mean ± SD.

The physicochemical parameters of different CSRW samples are shown in Figure 1. TA-1 exhibited the highest alcohol content (2.7%), which was significantly higher than that of other varieties, while HXR-450 showed the lowest alcohol content at 2.0% (Figure 1A). In contrast, HXR-450 displayed the highest total sugar content, whereas TA-1 exhibited the lowest (Figure 1B). Notably, the total sugar content of GR and the three aromatic japonica rice varieties was generally higher than that of the two non-aromatic japonica rice varieties, accompanied by a decrease in alcohol content. This difference may be attributed to variations in starch composition, gelatinization properties, and fermentable sugar release rates among different rice varieties. Total acid content directly affects the taste harmony of rice wine. CS-217 exhibited significantly higher total acid content than other varieties, while TA-1 showed the lowest total acid content, which may be related to the protein content of the different rice varieties (Figure 1C) [24]. Significant differences in pH values were also observed among different japonica rice varieties, with the HR-1212 group exhibiting the lowest pH value. These findings are consistent with a previous study, which also confirmed significant differences in the physicochemical indices of sweet rice wine fermented from different rice varieties [25].

**Figure 2:**
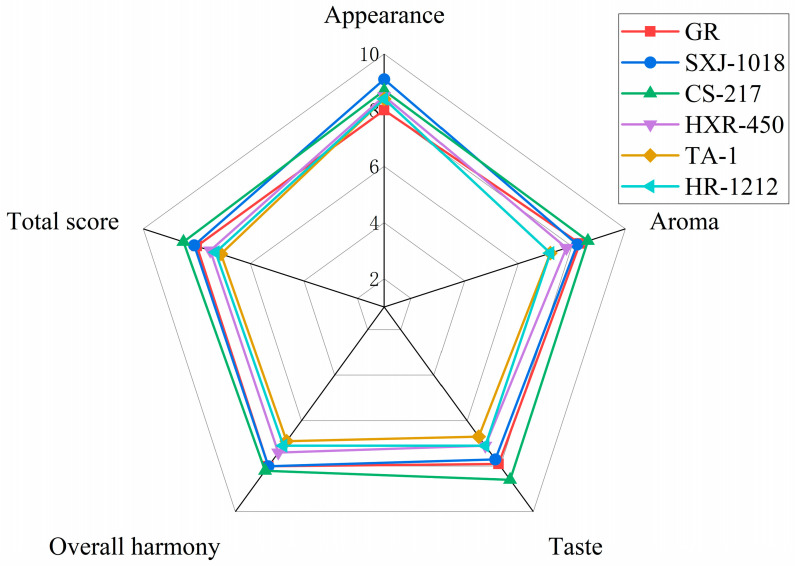
Sensory evaluation of different CSRW.

The sensory evaluation results of CSRW samples are shown in Figure 2. Significant differences in flavor and taste were observed among CSRW brewed from different rice varieties. CS-217 received the highest scores in aroma, taste, and overall harmony, indicating its superior overall sensory quality. In contrast, HXR-450, TA-1, and HR-1212 scored lower than glutinous rice in all evaluated attributes. HXR-450 exhibited a relatively light and less layered flavor, which may be attributed to its incomplete fermentation process and low alcohol content [26]. Similarly, the relatively low total acid and total sugar contents in TA-1 and HR-1212 may contribute to their less pronounced flavor characteristics.

### 3.2. Total Phenols, Flavonoid Contents, and Antioxidant Capacity

**Figure 3:**
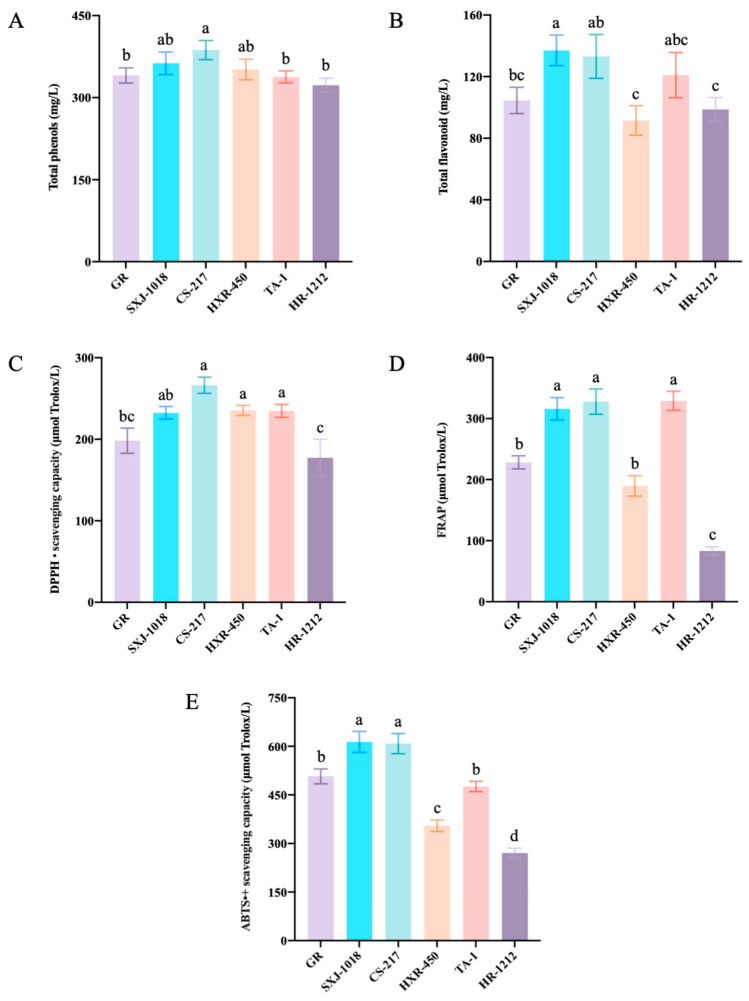
Total phenols (**A**), flavonoid (**B**) contents, DPPH• scavenging capacity (**C**), FRAP (**D**), and ABTS•+ scavenging capacity (**E**) in different CSRW. Error bars represent the standard deviation of the mean scores. Different letters mean significant differences between groups (*p*-value < 0.05), data are presented as mean ± SD.

Phenolic compounds, such as flavonoids and phenolic acids, are natural bioactive components in plants. Their concentrations are closely related to antioxidant activity due to their strong free radical scavenging capacity [21]. Figure 3A shows the total phenolic content (TPC) of CSRW brewed from different rice varieties, ranging from 322.76 to 387.08 mg/L. Among them, CS-217 exhibited the highest TPC (387.08 mg/L), followed by SXJ-1018, while HR-1212 showed the lowest TPC (322.76 mg/L). However, it should be noted that the Folin–Ciocalteu method responds to all reducible substances, and CSRW is rich in non-phenolic reductants such as reducing sugars and fermentation-derived metabolites, which may contribute to the measured TPC values. Thus, the reported TPC reflects total reducing capacity rather than absolute phenolic content in the present study. The total flavonoid content followed a similar trend, ranging from 91.55 to 137.05 mg/L, with SXJ-1018 and CS-217 exhibiting the highest flavonoid accumulation, and HXR-450 and HR-1212 showing the lowest contents (Figure 3B). This may be attributed to the phenolic and flavonoid compounds in sweet rice wine, which are primarily derived from the rice raw materials, and even under identical fermentation conditions and starter culture, different rice varieties can result in markedly different quality parameters in the final product [3,6].

In this study, the antioxidant activities of CSRW were comprehensively evaluated using DPPH and ABTS radical scavenging capacities as well as FRAP ferric reducing power (Figure 3C–E). The results showed significant differences in antioxidant capacity among CSRW brewed from different rice varieties, which is consistent with previous findings [3]. CS-217 exhibited outstanding performance in DPPH scavenging rate, ABTS inhibition rate, and FRAP reducing power, demonstrating the strongest overall antioxidant activity, which is closely related to its higher total phenolic and total flavonoid contents. Previous studies have confirmed a significant positive correlation between polyphenol content and DPPH and ABTS scavenging capacities [27]. In addition, FRAP values may also be associated with specific amino acids in sweet rice wine, as it has been reported that the radical absorption capacity of rice wine is positively correlated with tryptophan, cystine, methionine, and tyrosine [28]. In contrast, HR-1212 exhibited the lowest values in ABTS inhibition, DPPH scavenging, and Fe^3+^ reducing ability, consistent with its lowest total phenolic content.

### 3.3. HS-GC-IMS Analysis of Volatile Compounds in CSRW

#### 3.3.1. Determination of VOCs

**Figure 4:**
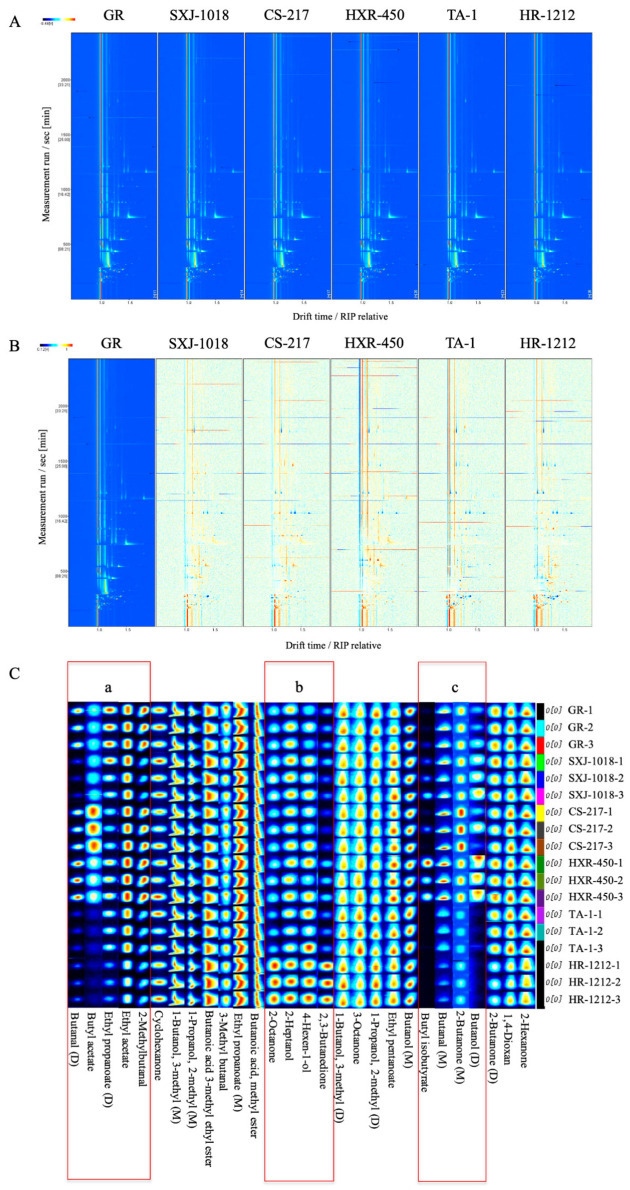
HS-GC-IMS analysis of CSRW fermented with diverse rice varieties; (**A**) topographic plots; (**B**) the difference comparison topographic plots; (**C**) fingerprint of volatile compounds: (**a**) higher signal intensities in the glutinous rice and aromatic japonica rice groups; (**b**) higher signal intensities in the non-aromatic japonica rice groups; (**c**) higher signal intensities in the aromatic japonica rice groups.

The VOCs of CSRW brewed from different rice varieties were analyzed using HS-GC-IMS. The retention index (RI), retention time (Rt), and drift time (Dt) were used for the qualitative identification of VOCs in the samples, while the signal intensity was employed for quantitative analysis [22]. As shown in Figure 4A,B, most signals occurred within a Dt range of 1.0–2.0 s and a Rt range of 200–900 s. Using the spectral data of GR as a reference for comparison with other CSRW samples, significant differences were observed among CSRW samples brewed from different rice varieties.

To identify the compounds responsible for these differences, fingerprint profiles of the VOCs were constructed for qualitative analysis (Figure 4C). A total of 28 VOCs were identified, including eight esters, seven ketones, eight alcohols, four aldehydes, and one other compound. Furthermore, fingerprint analysis revealed distinct patterns of VOCs among different CSRW samples. Region a was characterized by higher signal intensities in the glutinous rice and aromatic japonica rice groups, including ethyl acetate, butyl acetate, ethyl propanoate (D), butanal (D), and 2-methylbutanal, which constituted the typical fruity and sweet aroma base. Region b exhibited higher signal intensities in the non-aromatic japonica rice groups, including 2-octanone, 2-heptanol, 4-hexen-1-ol, and 2,3-butanedione, contributing to creamy, fatty, and herbaceous aromas. In Region c, butanol (D), butanal (M), 2-butanone (M), and butyl isobutyrate showed stronger signal intensities in the aromatic japonica rice groups. The fingerprint results were consistent with the spectral data, confirming that CSRW brewed from different rice varieties exhibited significant differences in VOC composition [9].

The formation of esters in rice wine is mainly attributed to esterase-mediated reactions between alcohols and acids derived from the microbial metabolism of rice starch, proteins, and fats, which collectively contribute to the characteristic pleasant aroma profile of the beverage [29,30]. Esters such as 3-methylbutyl butanoate (fruity), ethyl pentanoate (fruity), ethyl propanoate (M) (fruity, winery), and methyl butanoate (fruity, sweet) maintained relatively high signal intensities across all CSRW samples, serving as sources of the floral, fruity, and sweet aromas of sweet rice wine, and imparting pleasant flavor characteristics such as apple, pineapple, and banana to the wine. In CSRW samples made from glutinous rice and aromatic japonica rice, higher signal intensities were observed for esters associated with pronounced fruity characteristics, such as ethyl acetate (sweet, fruity), butyl acetate (sweet, floral, fruity), and ethyl propanoate (D) (fruity, winery), thereby enriching their sensory profiles of sweetness, fruitiness, winery notes, and floral aromas [31]. In addition, butyl isobutyrate (fruity, winery) exhibited higher signal intensity in aromatic japonica rice wine, enhancing its sweet, fruity, winery, and creamy sweetness characteristics.

Alcohols are an important component of the aroma profile of rice wine and significantly influence its overall flavor [6]. In this study, various alcohols, including 3-methyl-1-butanol (D), 2-methyl-1-propanol (D), and 1-heptanol, were detected in all CSRW samples, which is consistent with previous findings [32]. However, although the types of alcohols in CSRW are similar, their different concentrations may affect sensory perception, leading to differences in the taste and flavor of samples [33]. Aldehydes and ketones contribute to enhancing the flavor complexity of rice wine and coordinate the release of aromas [34]. A series of aldehydes and ketones were identified in the present study, including 2,3-butanedione (butter, cream, nutty), 2-methylbutanal (chocolate, coffee, malty), and 2-octanone (fruity, floral), among others. These compounds, together with esters and alcohols, not only enrich the aroma layers of sweet rice wine but also play an important role in influencing its taste and mouthfeel [35,36].

#### 3.3.2. Differential VOCs in CSRW Fermented with Diverse Rice Varieties

**Figure 5:**
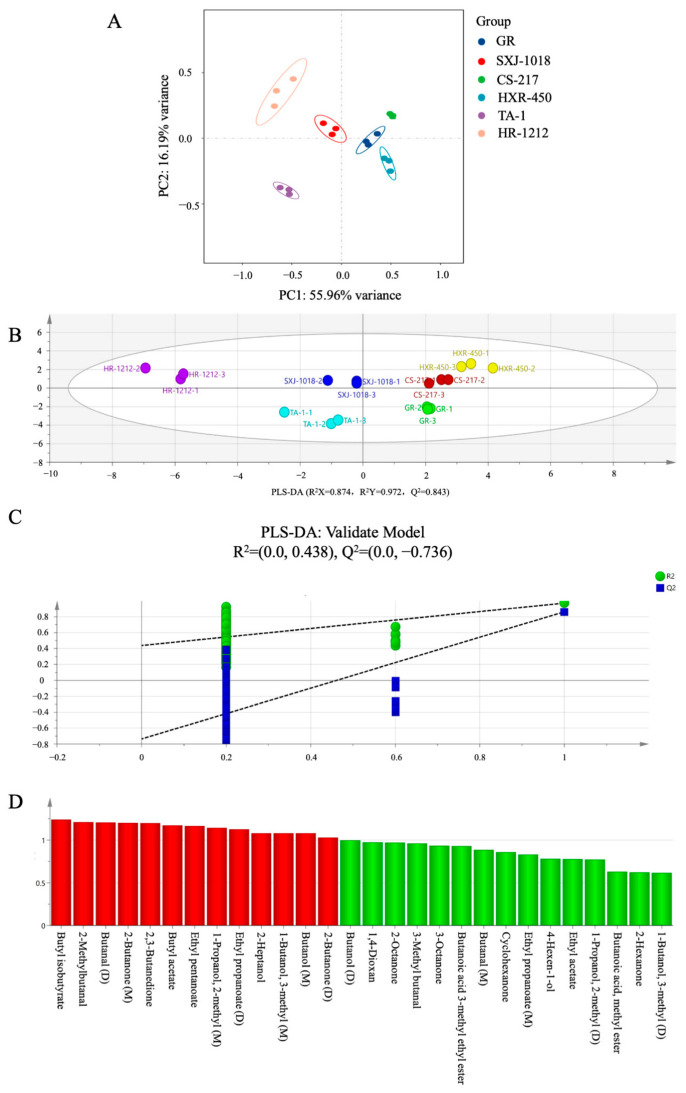
PCA (**A**), PLS-DA (**B**), permutation test (**C**), and VIP score (**D**) based on VOCs in CSRW.

Principal component analysis (PCA) was employed to reduce the dimensionality of the data for better identification of the impact of different rice varieties on the VOC profiles of CSRW (Figure 5A). The first two principal components, PC1 and PC2, accounted for 55.96% and 16.19% of the total variance, respectively, effectively explaining the majority of the VOCs information across the CSRW samples. Notably, a clear distinction was observed between the non-aromatic japonica rice group and the groups of glutinous rice and aromatic japonica rice. To further maximize inter-group differences and elucidate the relationship with sample categories, supervised partial least squares discriminant analysis (PLS-DA) was performed [37]. The PLS-DA model demonstrates superior analytical performance and effectiveness over PCA in precisely identifying key parameters influencing CSRW characteristics [38]. Using 28 VOCs as independent variables and the CSRW groups as the dependent variable, a PLS-DA model was established, with the resulting score plot and permutation test presented in Figure 5B,C. The model parameters (R^2^X = 0.874, R^2^Y = 0.972, Q^2^ = 0.843), validation results (R^2^ = 0.438, Q^2^= −0.736), and misclassification table (Table S1) confirm the reliability and robustness of the constructed PLS-DA model.

In addition, the PLS-DA model was employed to calculate the variable importance in projection (VIP) values of VOCs for predicting and evaluating their contributions to variations in CSRW [39]. VIP values were used to assess the differential contributions of VOCs in CSRW produced from different rice varieties, with VIP ≥ 1 serving as the criterion for significant contribution. Butyl isobutyrate, 2-methylbutanal, butanal (D), 2-butanone (M), 2,3-butanedione, butyl acetate, ethyl pentanoate, 1-propanol, 2-methyl (M), ethyl propanoate (D), 2-heptanol, 1-butanol, 3-methyl (M), butanol (M), and 2-butanone (D) were identified as key compounds responsible for discriminating VOCs among various CSRW samples (Figure 5D). These key compounds also contributed to the differences in sensory evaluation among different CSRW samples. The differences in compounds may be attributed to two reasons. First, inherent differences exist in the VOC composition of different rice varieties themselves. For instance, aromatic japonica rice naturally contains more characteristic aroma substances such as low-threshold aldehydes. These volatile components derived from raw materials can be partially retained during the fermentation process, directly participating in and shaping the overall aroma profile of CSRW, thereby leading to differences in volatile compounds among different CSRW samples [12,16]. Second, rice varieties differ in protein content, starch structure, and lipid content, which can indirectly affect microbial growth and metabolic activities, potentially generating alcohol, aldehyde, ketone, and ester precursors with varying types and abundances, consequently leading to differences in the final VOCs of CSRW [12].

#### 3.3.3. ROAV

ROAV is a widely used method for identifying key flavor compounds in foods. By considering the sensory threshold of each compound, ROAV quantifies the contribution of individual volatile compounds to the overall aroma profile of samples [23]. Compounds with ROAV ≥ 1 are defined as those that directly influence the flavor of samples, whereas those with 0.1 ≤ ROAV < 1.0 are regarded as important contributors in modulating the overall aroma [40]. In the present study, ethyl pentanoate was assigned an ROAV value of 100 due to its relatively low sensory threshold and high signal intensity detected in HXR-450. The ROAVs of the other nine key compounds were calculated based on reported sensory thresholds to evaluate their contributions to the flavor of CSRW (Table 1). Calculation results showed that 2-methylbutanal, butanal (D), 2,3-butanedione, butyl acetate, ethyl pentanoate, and 2-heptanol exhibited ROAV ≥ 1 in all CSRW samples, contributing complex aromas such as fruity, buttery, nutty, and alcoholic notes to the wine.

### 3.4. UPLC-MS/MS Analysis of Non-Volatile Metabolites

#### 3.4.1. Classification of Non-Volatile Metabolites

**Figure 6:**
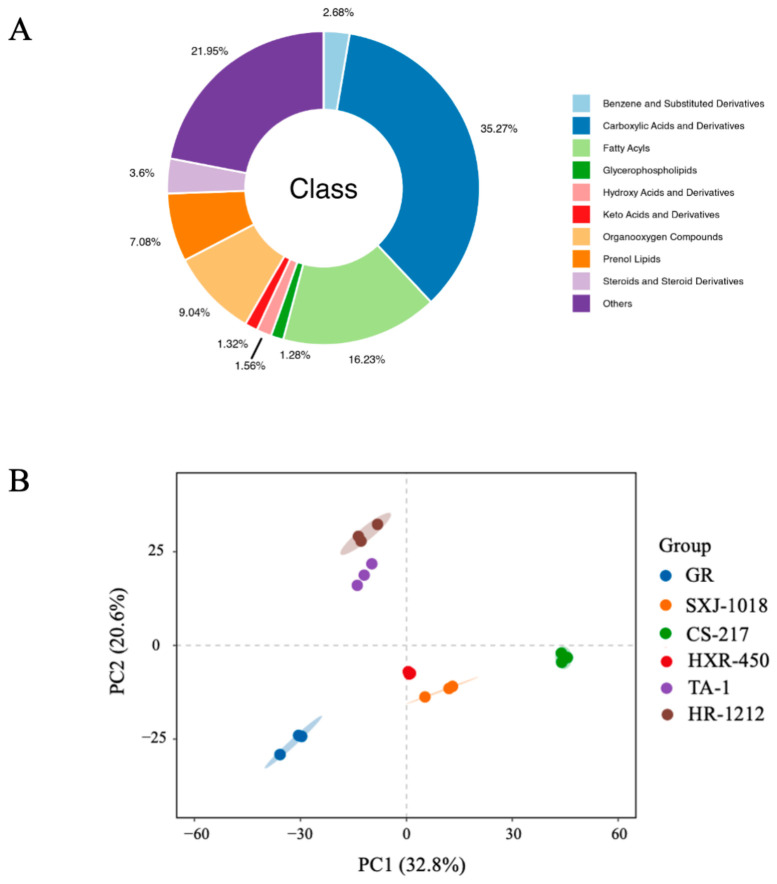
Metabolite categories (**A**) and PCA (**B**) based on non-volatile metabolites in CSRW.

Non-volatile metabolites in CSRW were analyzed and identified by UPLC-MS/MS, and a total of 2501 compounds were characterized (Table S2). These compounds were mainly classified into carboxylic acids and derivatives, fatty acyls, organic oxygen compounds, prenol lipids, benzene and substituted derivatives, steroids and steroid derivatives, and others (Figure 6A). Among them, carboxylic acids and derivatives showed the highest relative content in CSRW, followed by fatty acyls. A PCA plot based on the relative abundance of metabolites was generated, in which the PC1 and PC2 explained 53.4% of the metabolic variance, revealing distinct metabolic differences among CSRW samples (Figure 6B). Specifically, SXJ-1018 and HXR-450 derived from aromatic japonica rice clustered closely, indicating small compositional differences between them. Similarly, TA-1 and HR-1212 from non-aromatic japonica rice were also positioned together. In contrast, GR and CS-217 exhibited greater compositional differences from other samples, and the largest compositional difference was observed between GR and CS-217.

**Figure 7:**
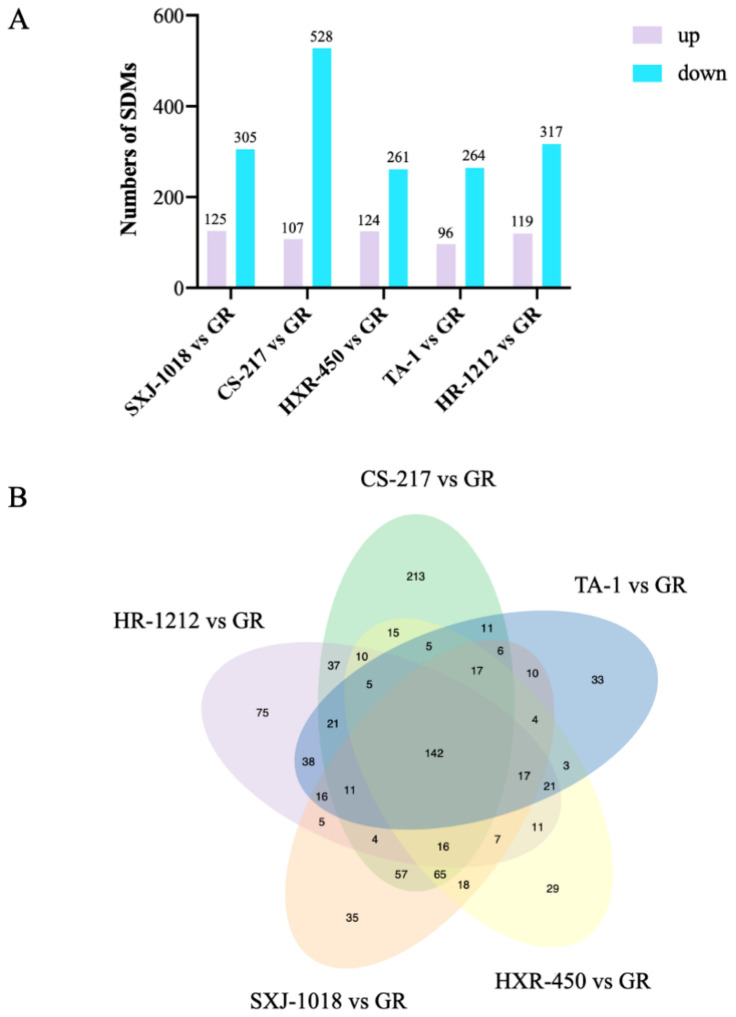
Quantification and overlap of SDMs in CSRW: (**A**) number of SDMs and (**B**) Venn diagram analysis.

Significantly differential metabolites (SDMs) were screened based on fold change (FC) ≥ 2 or ≤0.5, and *p* < 0.05 (Figure 7A and Figure S1). The results showed that there were 430 (125 upregulated, 305 downregulated), 635 (107 upregulated, 528 downregulated), 385 (124 upregulated, 261 downregulated), 360 (96 upregulated, 264 downregulated), and 436 (119 upregulated, 317 downregulated) SDMs between SXJ-1018, CS-217, HXR-450, TA-1, HR-1212, and GR, respectively.

A Venn diagram (Figure 7B) illustrated the overlapping relationships of SDMs across different comparison groups. Comparisons of SXJ-1018, CS-217, HXR-450, TA-1, and HR-1212 against GR revealed 35, 213, 29, 33, and 75 unique SDMs, respectively. These unique metabolites may be related to the inherent compositional differences in starch structure, metabolites, and proteins among diverse rice varieties [10,46]. In addition, 142 common SDMs were identified across the different comparison groups, which may contribute to the similarities among various sweet rice wines in terms of flavor and taste.

#### 3.4.2. Metabolite Pathway Enrichment Analysis

**Figure 8:**
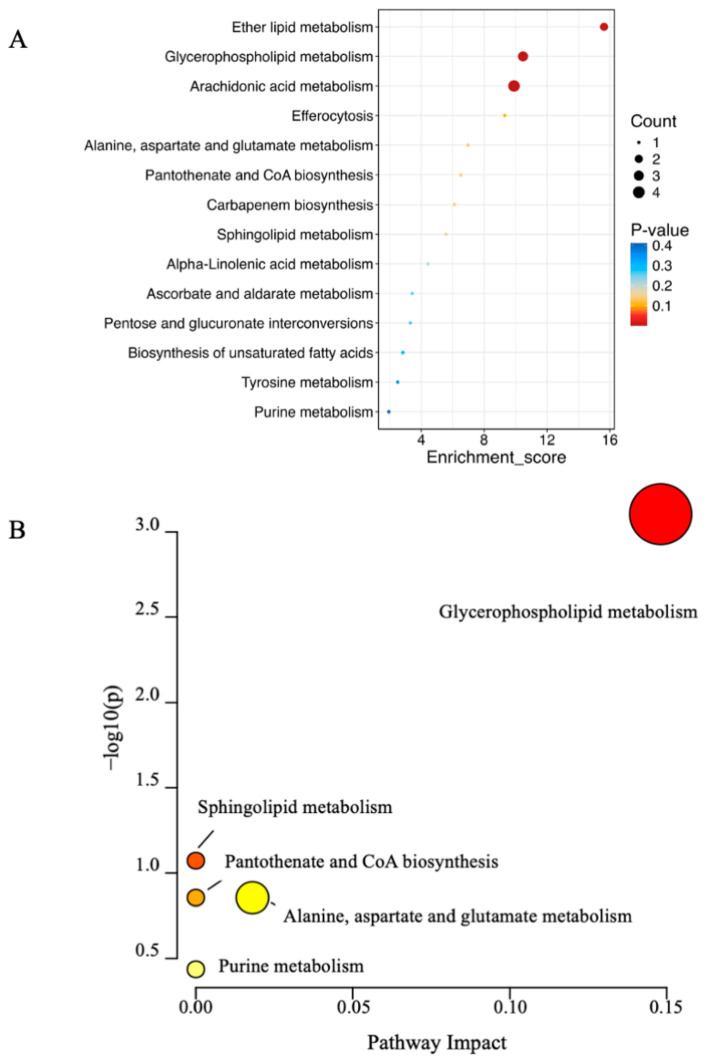
Metabolic pathway enrichment analysis of common SDMs in CSRW: (**A**) enriched KEGG pathways and (**B**) key pathways identified via MetPA.

Different raw materials significantly influence the flavor and taste of rice wine [9,47]. Analysis of metabolic pathways may help elucidate the effects of raw materials on the functionality and quality of CSRW. Metabolic pathway analysis was performed using the KEGG database on 142 common SDMs from different comparison groups, highlighting the top-ranked metabolic pathways (Figure S2 and Figure 8A). Among these pathways, nine were consistently identified across all groups, including α-linolenic acid metabolism, tryptophan metabolism, sphingolipid metabolism, carbapenem biosynthesis, alanine, aspartate, and glutamate metabolism, glycerophospholipid metabolism, pantothenate and CoA biosynthesis, ether lipid metabolism, and arachidonic acid metabolism.

Tryptophan metabolism and alanine, aspartate, and glutamate metabolism belong to amino acid metabolism. Among them, alanine, aspartate, and glutamate metabolism may not only provide essential amino acids and nitrogen sources for the rapid growth of yeast and Rhizopus, but also potentially produce glutamic acid and aspartic acid, which are important umami amino acids in CSRW [48]. Furthermore, glutamic acid is known to positively enhance perceived taste while suppressing undesirable tastes such as sourness and bitterness [49]. As an important aromatic amino acid, tryptophan can be metabolized to generate alcohol and indole flavor precursors, which may contribute to the aroma layers and flavor complexity of sweet rice wine, and tryptophan is also associated with the formation of antioxidants [37,50]. Ether lipid metabolism, glycerophospholipid metabolism, and sphingolipid metabolism are lipid metabolic pathways. Lipid metabolism is involved in various cellular processes in yeast, including cell division, sporulation, apoptosis, and stress response [51]. Meanwhile, lipids are known to influence yeast performance and the production of aromatic compounds in fermented rice wine [52]. Pantothenate and CoA biosynthesis is a key pathway in sweet rice wine brewing, as the synthesis of many pleasant fruity and floral esters (such as acetates) requires acetyl-CoA as a direct substrate [53]. In addition, glycolysis and the TCA cycle are dependent on CoA, which also affects the rate and yield of alcohols and basic flavor compounds [37]. The α-linolenic acid and arachidonic acid metabolism belong to unsaturated fatty acid metabolic pathways. α-Linolenic acid is present in rice and can be catalytically oxidized by lipoxygenase to form compounds such as hexanal and hexanol, as well as β-oxidation to produce hexanoic acid and butyric acid [54,55]. Arachidonic acid is mainly present in microbial cell membranes and can generate hydroperoxyeicosatetraenoic acid (HPETE) through the lipoxygenase (LOX) pathway, which is further converted into volatile short-chain aldehydes, ketones, and alcohols [56]. All these metabolic pathways may be collectively associated with the quality formation and flavor characteristics of CSRW.

**Figure 9:**
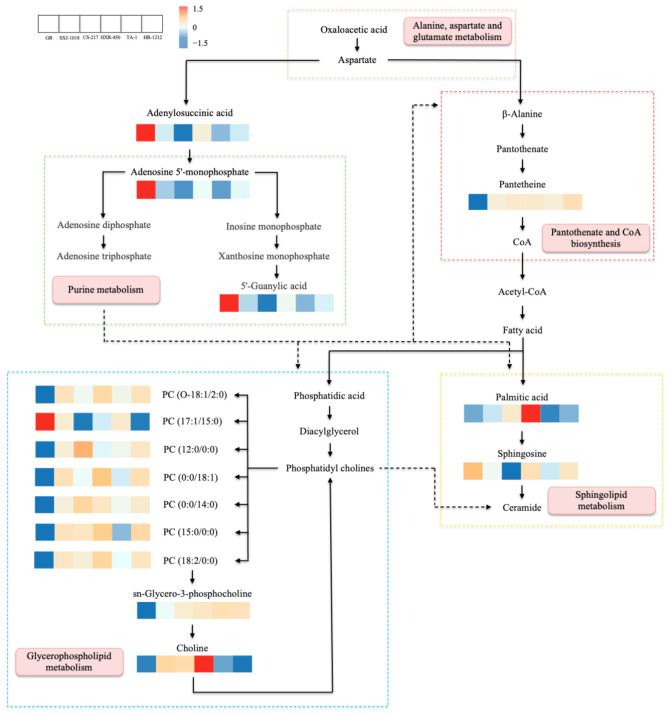
Key metabolic pathway network plots of CSRW. Metabolite abundance levels are represented as a heatmap within the network plot.

MetPA is an analytical method based on the KEGG database that combines betweenness centrality and the hypergeometric test to identify crucial metabolic pathways [57]. As shown in Figure 8B, five pathways were identified by MetPA analysis, including sphingolipid metabolism, pantothenate and CoA biosynthesis, alanine, aspartate and glutamate metabolism, glycerophospholipid metabolism, and purine metabolism. The metabolic interactions among these five pathways are illustrated in Figure 9. Aspartate participates in two key biosynthetic branches. On one hand, it reacts with inosine monophosphate (IMP) to form adenylosuccinate, which enters the purine metabolism pathway to synthesize adenosine monophosphate (AMP) and ultimately generate ATP. ATP serves as the universal energy currency driving biosynthesis and is also an important precursor for the synthesis of guanosine monophosphate (GMP), a potent umami enhancer [58,59]. On the other hand, aspartate is decarboxylated to β-alanine, initiating the pantothenate and CoA biosynthesis pathway and ultimately yielding coenzyme A (CoA) [60]. Acetyl-CoA, the activated form of CoA, acts as the universal precursor for fatty acid biosynthesis. The resulting fatty acids provide essential acyl chains for two major membrane lipid-synthetic pathways: glycerophospholipid metabolism and sphingolipid metabolism. The former generates structural lipids such as phosphatidylcholine and phosphatidylethanolamine to maintain membrane integrity, while the latter synthesizes signaling lipids such as sphingosine and palmitoyl ceramide, which are critical for signal transduction and membrane domain organization [61]. These interconnected metabolic pathways may collectively contribute to the flavor characteristics of CSRW.

## 4. Conclusions

This study investigated the impact of different rice varieties on the flavor and metabolic characteristics of CSRW by evaluating physicochemical indicators, VOCs, and metabolites. The results showed that CSRW brewed from non-aromatic japonica rice had significantly higher alcohol content than that from glutinous rice, though with lower total sugar levels. CS-217 exhibited the highest total acid, total phenolic content, and pH, along with relatively higher ABTS inhibition rate, DPPH scavenging capacity, and Fe^3+^-reducing capacity compared to other groups, and demonstrated superior overall sensory evaluation. A total of 28 VOCs were identified. Among them, ethyl acetate, butyl acetate, and ethyl propanoate (D) exhibited higher signal intensities in CSRW made from glutinous rice and aromatic japonica rice, while butyl isobutyrate exhibited higher signal intensity exclusively in aromatic japonica rice wines. Thirteen volatile compounds, including butyl isobutyrate, butyl acetate, ethyl pentanoate, 1-propanol, 2-methyl (M), and ethyl propanoate (D), were identified as key discriminators of VOC profiles among the various sweet rice wines. ROAV results indicated that 2-methylbutanal, butanal (D), 2,3-butanedione, butyl acetate, ethyl pentanoate, and 2-heptanol contributed significantly to the flavor of CSRW. UPLC-MS/MS analysis identified 2501 non-volatile metabolites in CSRW, with carboxylic acids and derivatives being the most abundant. MetPA analysis based on the 142 shared SDMs identified five key metabolic pathways, primarily including Purine metabolism and Pantothenate and CoA biosynthesis. In summary, CSRW produced from different rice varieties exhibited notable differences in flavor and taste. This study provides new insights for diversifying raw materials in CSRW production.

## Figures and Tables

**Table 1:** Odor threshold and ROAV of differential VOCs in CSRW.

VOC	Odor Threshold (μg/L)	ROAV					
		GR	SXJ-1018	CS-217	HXR-450	TA-1	HR-1212
2-methylbutanal	89.47 ^a^	76.43	35.24	70.77	82.44	42.44	41.80
Butanal (D)	17 ^b^	30.90	9.00	28.03	38.38	9.73	11.80
2,3-butanedione	20.44 ^c^	24.75	22.48	15.48	30.07	22.18	67.95
Butyl acetate	86.95 ^a^	6.87	6.53	12.67	8.49	2.50	1.99
Ethyl pentanoate	26.8 ^d^	79.70	97.24	97.03	100.00	93.07	98.09
1-propanol, 2-methyl (M)	11,673.24 ^a^	1.01	1.13	1.16	1.18	0.97	1.18
Ethyl propanoate (D)	1800 ^e^	0.47	0.38	0.28	0.37	0.33	0.20
2-heptanol	61.61 ^a^	17.14	18.71	16.55	17.81	14.74	20.70
1-butanol, 3-methyl (M)	404,582 ^a^	<0.1	<0.1	<0.1	<0.1	<0.1	<0.1
Butanol (M)	150,000 ^f^	<0.1	<0.1	<0.1	<0.1	<0.1	<0.1

^a^ Odor thresholds taken from reference [41]. ^b^ Odor thresholds taken from reference [42]. ^c^ Odor thresholds taken from reference [22]. ^d^ Odor thresholds taken from reference [43]. ^e^ Odor thresholds taken from reference [44]. ^f^ Odor thresholds taken from reference [45].

## Data Availability

The original contributions presented in the study are included in the article/Supplementary Materials, further inquiries can be directed to the corresponding authors.

## Supplementary Materials

The following supporting information can be downloaded at https://www.mdpi.com/article/10.3390/foods15122137/s1: Figure S1. Volcano plots of SDMs in CSRW: (A) SXJ-1018 vs. GR, (B) CS-217 vs. GR, (C) HXR-450 vs. GR, (D) TA-1 vs. GR, (E) HR-1212 vs. GR; Figure S2. Differential metabolite enrichment pathway analysis: (A) SXJ-1018 vs. GR, (B) CS-217 vs. GR, (C) HXR-450 vs. GR, (D) TA-1 vs. GR, (E) HR-1212 vs. GR; Table S1. Misclassification table of PLS-DA model; Table S2. Screening and classification of metabolites in CSRW.
