# Supplementary material for: Revealing the Flavor and Metabolite Differences of Chinese Sweet Rice Wine Fermented with Diverse Rice Varieties Using GC-IMS and UPLC-MS/MS
Source: Foods. 2026 Jun 13;15(12):2137. doi: 10.3390/foods15122137 (PMC13298584; doi:10.3390/foods15122137)
Supplement: Supplementary file 1 [file foods-15-02137-s001.zip › supplementary materials Figures.pdf]

Supplementary materials

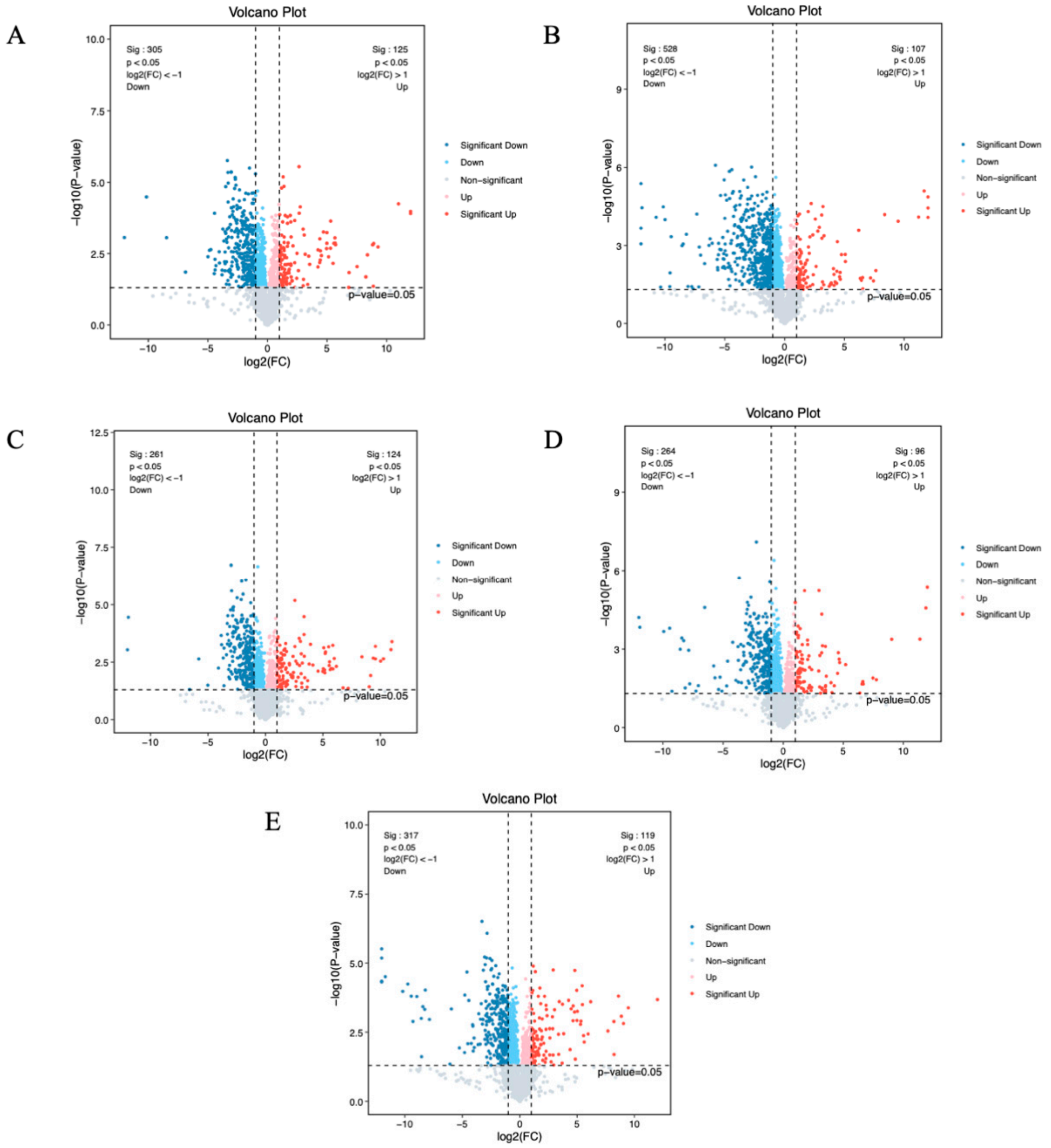

Figure S1. Volcano plots of SDMs in CSRW: (A) SXJ-1018 vs. GR, (B) CS-217 vs. GR, (C) HXR-450 vs. GR, (D) TA-1 vs. GR, (E) HR-1212 vs. GR.

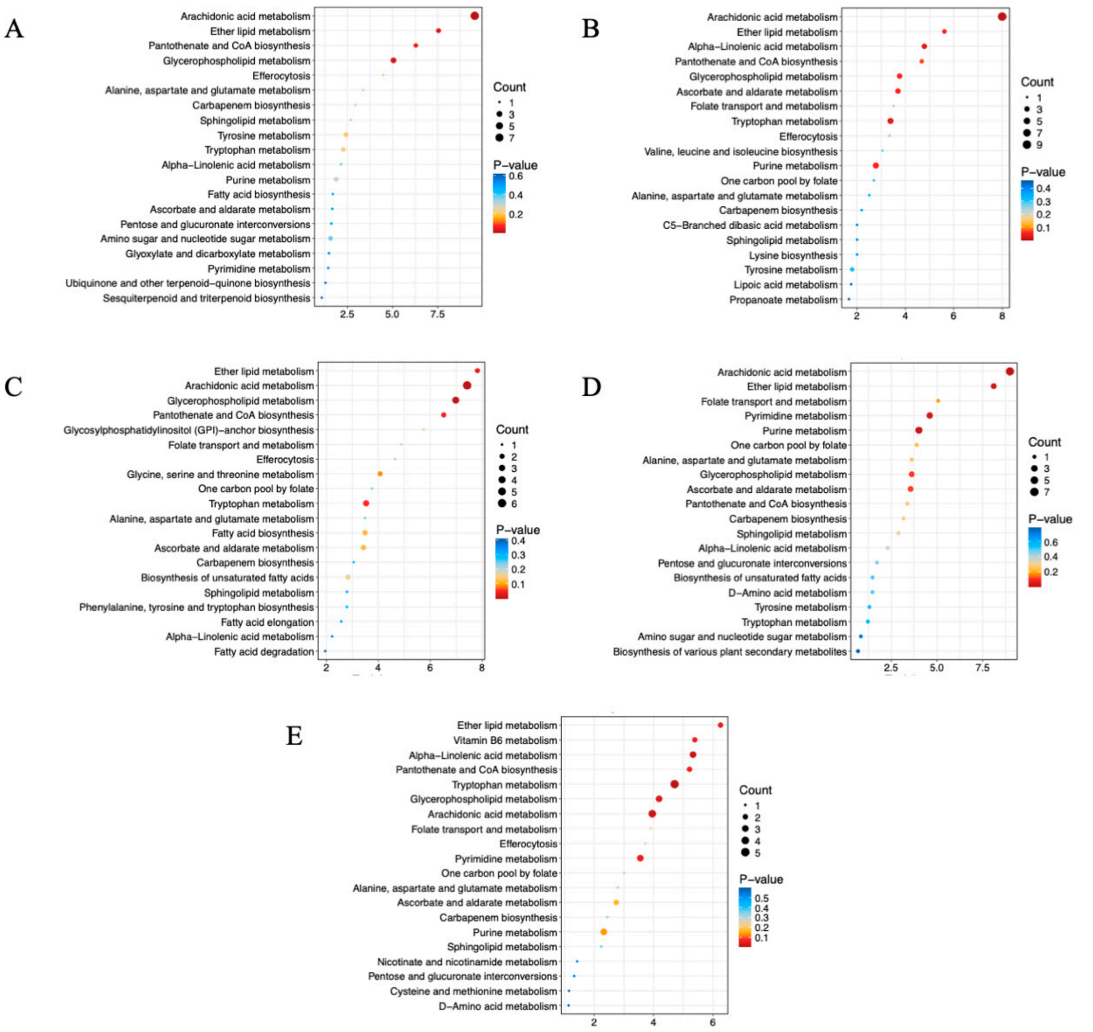

Figure S2. Differential metabolite enrichment pathway analysis: (A) SXJ-1018 vs. GR, (B) CS-217 vs. GR, (C) HXR-450 vs. GR, (D) TA-1 vs. GR, (E) HR-1212 vs. GR.
