# Supplementary material for: Revealing the Flavor and Metabolite Differences of Chinese Sweet Rice Wine Fermented with Diverse Rice Varieties Using GC-IMS and UPLC-MS/MS
Source: Foods. 2026 Jun 13;15(12):2137. doi: 10.3390/foods15122137 (PMC13298584; doi:10.3390/foods15122137)
Supplement: Supplementary file 1 [file foods-15-02137-s001.zip › Table S1.pdf]

Table S1. Misclassification table of PLS-DA model

Group	Correct (%)	GR	SXJ-1018	CS-217	HXR-450	TA-1	HR-1212	No class (YPred ≤ 0)
GR	100%	3	0	0	0	0	0	0
SXJ-1018	100%	0	3	0	0	0	0	0
CS-217	100%	0	0	3	0	0	0	0
HXR-450	100%	0	0	0	3	0	0	0
TA-1	100%	0	0	0	0	3	0	0
HR-1212	100%	0	0	0	0	0	3	0
No Class	0%	0	0	0	0	0	0	0
Total	100%	3	3	3	3	3	3	0
Fisher's prob.					5.2e-09			
